# P-521. The Many Faces of Pediatric Long COVID: A Phenotypic Cluster Analysis of Symptoms in Children Presenting to a Multidisciplinary Pediatric Long COVID Clinic

**DOI:** 10.1093/ofid/ofaf695.736

**Published:** 2026-01-11

**Authors:** Alexandra B Yonts, Pallavi Dwivedi, James Bost, Monika Geslak, Erin McLaughlin, Emily Ansusinha

**Affiliations:** Children's National Hospital/ George Washington University, Washington, District of Columbia; Children’s National Hospital, Silverspring, Maryland; Children's National Health System, Washington, DC; Children's National Hospital, Washington, District of Columbia; George Washington University, Washington, District of Columbia; Children's National Hospital, Washington, District of Columbia

## Abstract

**Background:**

Long COVID is a complex infection-associated chronic condition that affects 10-25% of children after SARS-CoV-2 infection. Multiple underlying pathophysiologic mechanisms, including persistence of viral antigens, aberrant immune response, and endovascular dysfunction, are suspected to contribute to the heterogeneity of symptoms associated with this condition. Some symptoms (i.e. fatigue) are present in nearly all patients, while others appear to present in clusters, suggesting different pathophysiologic processes within subpopulations.

**Figure:**
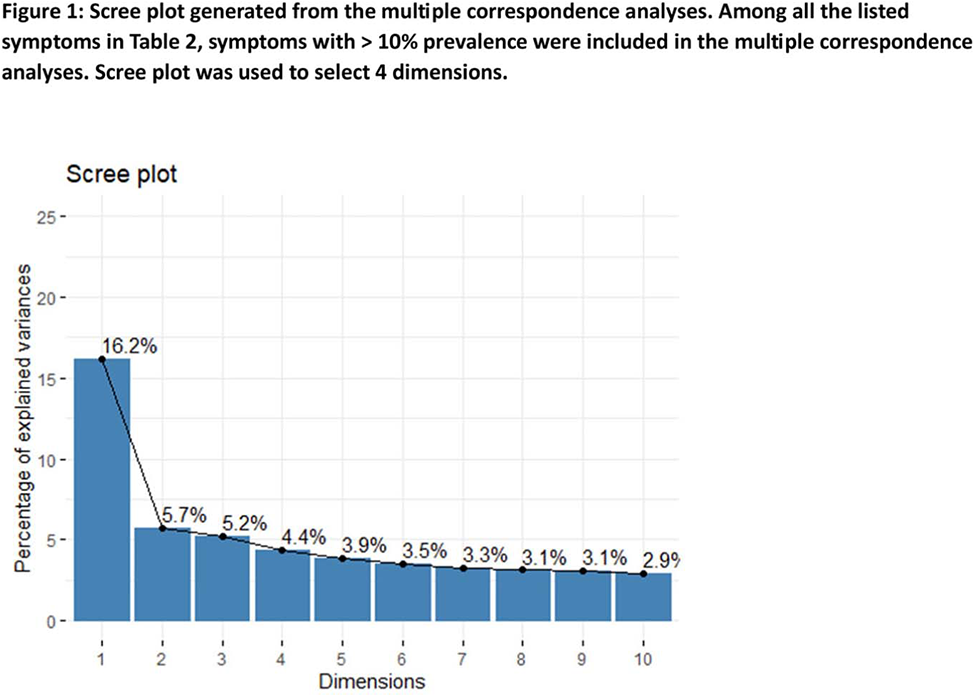

**Figure:**
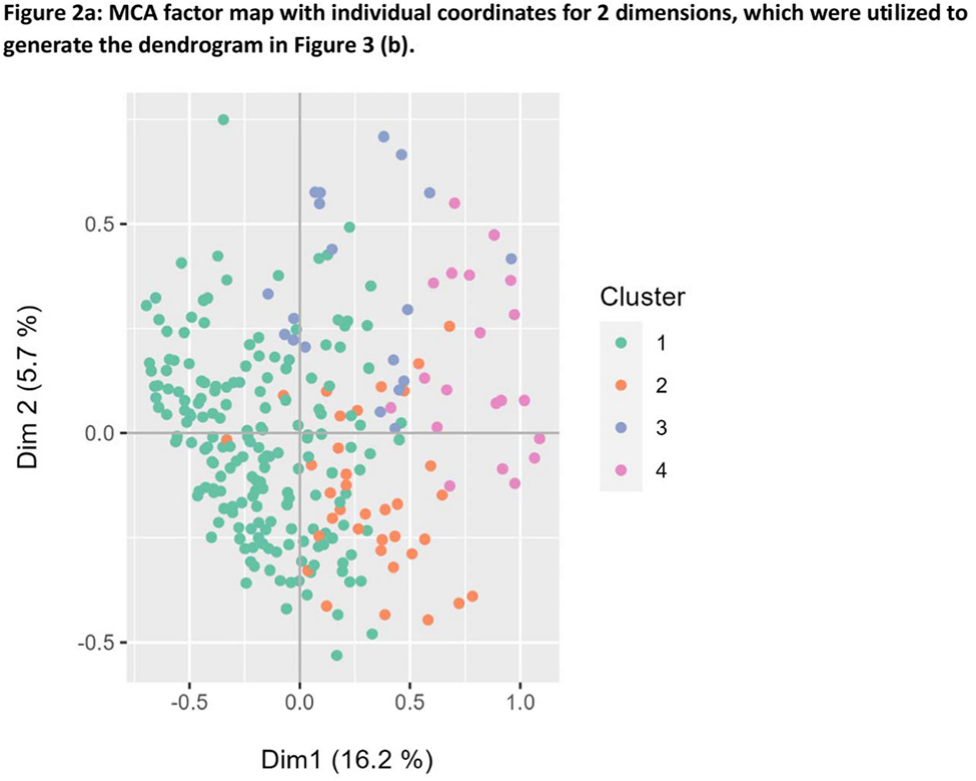

**Methods:**

In this retrospective study, we reviewed the clinical and demographic data of 254 patients seen in the Post COVID Program Clinic from May 2021- January 2024. We determined the frequency of 48 symptoms reported by patients at intake. Multiple Correspondence Analysis (MCA) was used to reduce dimensionality and generate groups of patients with similar symptom profiles. MCA analyses with average-linking clustering of MCA coordinates was conducted on symptoms present in > 10% of the cohort. Chi square or Fisher’s exact test were used to identify statistically significant patterns in the cohort.

**Figure:**
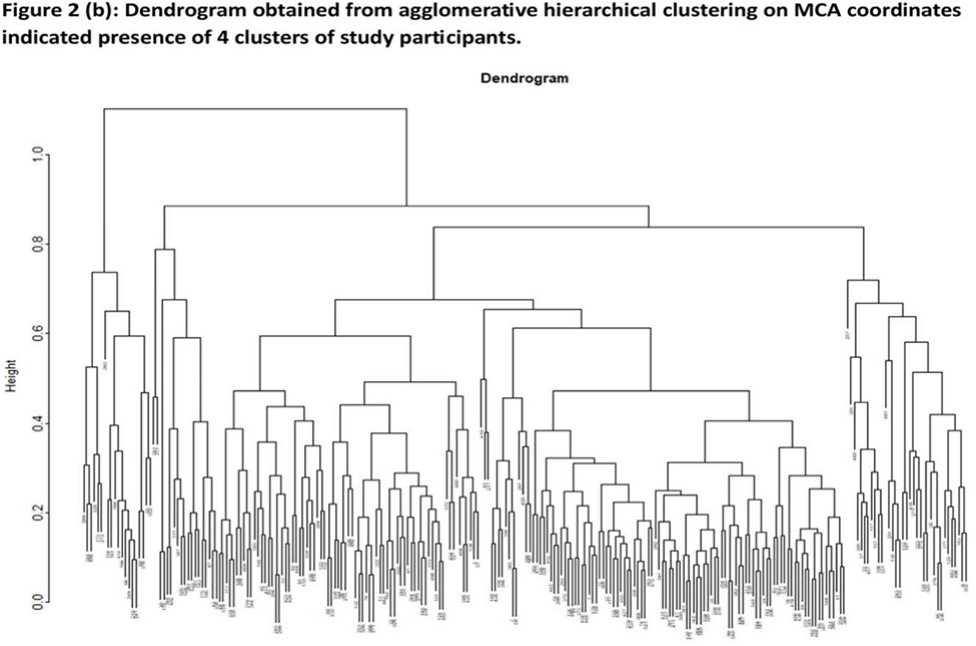

**Results:**

In 254 pediatric patients (56% female, average age 12.9 years [range 2-20]), the most frequently reported symptoms at the time of clinic intake (median 317 days after acute SARS-CoV-2 infection) were fatigue (91%), decreased exercise tolerance (77%), headache (75%), cognitive dysfunction (67%) and lightheadedness (61%). MCA revealed 4 symptomatic clusters. Two clusters, representing 13% (Cluster2) and 7% (Cluster3) of patients, were characterized by higher incidence of gastrointestinal, musculoskeletal and neurologic symptoms (C2) and cardiopulmonary symptoms (C3). Cluster4 patients (7%) presented with high incidence of symptoms of an active inflammatory process, including fever, night sweats, cough and diarrhea. C2 and C3 patients were slightly older on average (average 14 vs 12.9 years) and C3 was disproportionately female (89% vs 56%).

**Conclusion:**

Children and adolescents with Long COVID may present with distinct phenotypes, potentially informing underlying processes and ideal management. Research and clinical evaluation of these clusters is in progress.

**Disclosures:**

Alexandra B. Yonts, MD, Pfizer: Grant/Research Support

